# Dogs’ olfactory resting-state functional connectivity is modulated by age and brain shape

**DOI:** 10.1038/s41598-025-95123-6

**Published:** 2025-04-15

**Authors:** Asami Nakaimuki, Bernadett Paska, Laura V. Cuaya, Raúl Hernández-Pérez, Kalman Czeibert, Dóra Szabó, Eniko Kubinyi, Attila Andics

**Affiliations:** 1 Department of Ethology, Institute of Biology, Eötvös Loránd University, Budapest, Hungary; 2 Doctoral School of Biology, Faculty of Science, Eötvös Loránd University, Budapest, Hungary; 3 MTA- ELTE NAP Canine Brain Research Group, Budapest, Hungary; 4 Social, Cognitive and Affective Neuroscience Unit, Department of Cognition, Emotion, and Methods in Psychology, Faculty of Psychology, University of Vienna, Vienna, Austria; 5 LimesVet Ltd, Budapest, Hungary; 6 MTA-ELTE Lendület “Momentum” Companion Animal Research Group, Budapest, Hungary

**Keywords:** Resting-state fMRI, Functional connectivity, Olfaction, Individual differences, Dog, Cognitive neuroscience, Olfactory system

## Abstract

Humans have long applied canine olfaction in various contexts. Dog olfactory brain networks have recently been mapped by anatomical measures, but functional connections remain unexplored. Also, whereas individual characteristics, including age, sex, and brain shape, are known to affect olfactory performance, their covariation with olfactory functional networks is unknown. To address these, we investigated dogs’ resting-state functional connectivities between anatomically defined olfactory regions and assessed whether and how their olfactory functional network is affected by age, sex, and brain shape. Olfactory functional connectivity strength exhibited negative correlations with both age and brain shape: older dogs and those with rounder-shaped brains demonstrated lower functional connectivity, respectively, but no effect of sex was found. The results suggest that both aging and brain morphology can negatively impact a dog’s sense of smell, and older dogs and dogs with rounder-shaped brains may have diminished olfactory performance.

## Introduction

Dogs have excellent olfactory abilities, which may be attributed to both the anatomical structure of their nose^1 ^and the genetic attributes^2^ of their olfactory system. Due to the dog’s high trainability and cooperative interaction with humans, as well as their olfactory sensitivity, canine olfaction has been utilized to serve humans in diverse contexts for a long time. Dogs greatly vary in their olfactory performance^3–6^. This variability is partly explained by individual characteristics, including breed^4,5^, brain shape^4,7^, age^6,8^, and sex^6,9^. Such variability is also observed in other species, including rodents (e.g.,^10,11^), non-human primates (e.g.,^12,13^), or humans (e.g.,^14,15^). Individual variability in the canine olfactory sensory system has been examined through various characteristics, including genetics (e.g.,^16^), skull morphology (e.g.,^17^), and brain anatomy (e.g.,^18^), but the correlation with brain function has not yet been explored.

A recent anatomical study with diffusion tensor imaging described the dog olfactory network^19^. This network consists of the olfactory bulb, the olfactory peduncle, the piriform cortex, the entorhinal cortex, the medulla oblongata, the occipital pole, the stria terminalis, and the orbito-frontal cortex. Most of this set of regions have been reported across evolutionarily distant mammals, including rodents^20,21 ^and humans^22^. The involvement of these regions in olfaction is also supported by dog functional magnetic resonance imaging (fMRI) works^23,24^.

In humans, it is widely recognized that individual differences such as sex and age influence the functional connectivity among olfactory brain regions^25–27^. Resting-state functional connectivity is often applied to investigate the difference in the fundamental functional brain networks based on individual characteristics, most frequently by pathological aspects^28^. However, while the dog’s resting-state functional connectivity involving the olfactory bulb and the piriform cortex has been explored^29^, a comprehensive investigation focusing on the entire olfactory network has not yet been attempted.

To assess anatomical correspondence with functional connectivity patterns and the effects of individual characteristics on functional connectivities, first, we explored functional connectivity in the anatomically defined olfactory network of dogs^19^. Subsequently, we examined the effects of sex, age, and brain shape on functional connectivities. We predicted that if there is a high level of anatomical-functional connectivity correspondence in the dog olfactory network, then functional connections will be stronger between olfactory regions that are also known to be more strongly connected by white-matter tracts. We also expected that if individual differences in olfactory abilities are reflected in network properties, then olfactory functional connectivities will be affected by individual characteristics such as age, sex, and brain shape, similarly to performance measures.

## Materials and methods

### Dataset

We utilized a previously collected resting-state functional MRI (fMRI) dataset^30^, comprising 33 family dogs (*Canis familiaris*). The data collection procedure of this dataset was approved by the Ethical Committee of Eötvös Loránd University (KA-1719/PEI/001/1490-4/2015) and by the Government Office of Pest County Directorate of Food Chain Safety and Animal Health (XIV-I-001/520-4/2012) and conducted in accordance with relevant guidelines and regulations. Owners provided informed consent and could stop the procedure at any time. Subjects were specifically trained to fMRI measurements, and the fMRI scanning was conducted without anesthesia under the condition that dogs could freely leave the scanner bed at any time^30,31^. The average subjects’ age was 6.79 ± 3.62 years (mean ± SD) between 1 and 14 years old, and the dataset contained 16 males and 17 females, representing 14 different breeds: nine Border Collies, seven Golden Retrievers, five Mongrels, two Australian Shepherds, one English Cocker Spaniel, one Labrador Retriever, one Labradoodle, one Mudi, one Swiss Shepherd, one Tervueren, one Springer Spaniel, one Chinese Crested dog, one Cairn Terrier, one Hungarian Vizsla. Images were acquired by a Siemens Prisma 3T scanner (Siemens Healthcare, Erlangen, Germany). The resting-state fMRI data were collected using a Gradient-Echo Echo-Planar Imaging (GRE-EPI) sequence (TR = 2640 ms, including a 500-ms delay at the end of each volume, TE = 31 ms, in-plane Field-of-View (FOV) = 128 mm × 128 mm with 2 mm in-plane resolution and slice thickness, 28 slices with 0.5 mm inter-slice gap, flip angle (FA) = 86°, and total acquisition time = 362 s/run). The phase-encoding direction was from the foot to the head side. For signal detection, a single loop coil (diameter = 11 cm) was applied. Each run lasted 6 min, containing 137 volumes, and was acquired between May 2018 and July 2019. The number of runs per subject ranged from two to five, 87 runs in total.

T1-weighted anatomical images were separately acquired for other studies by a 3T Philips Ingenia scanner (Philips Medical Systems, Best, The Netherlands), using a 3D Turbo Field Echo (TFE) sequence (TR = 9.85 ms, TE = 4.6 ms, and an isotropic resolution of 1 mm).

### Data preprocessing

Eighty-seven runs were applied for data processing. The preprocessing procedure included the following steps: (1) initial run selection based on head motion during scanning, using thresholds of 3 mm for translation and 2° for rotation; (2) realignment with six parameters; (3) reorientation of the brain image from the dog’s to the human’s orientation; (4) normalization to an in-house dog template brain image^32^, using previously normalized own T1-weighted images to the template, employing rigid registration and B-spline transformation; (5) origo setting, which is adjustment of the origin to align the normalized functional image with the template; (6) volume censoring based on framewise displacement (FD) with 50 mm in radius and temporal derivative RMS (root mean square) valence over voxels (DVARS), with thresholds set at 0.5 mm and 1.5%, respectively; (7) secondary run selection based on the number of remaining volumes after volume censoring per run; (8) a linear detrending; and (9) band-pass filtering with frequencies ranging 0.008–0.09 Hz. For these processes, MATLAB (version R2023b, MathWorks, Natick, MA, USA) (for steps 6–7), Statistical Parametric Mapping 12 (SPM12) software with MATLAB (Wellcome Centre for Human Neuroimaging, UCL, London, UK) (for steps 1–5), and the CONN functional connectivity toolbox with MATLAB (version 22.a, www.nitrc.org/projects/conn) (for steps 8–9) were applied. We did not apply spatial smoothing because it could average signals across voxels, potentially diminishing significant signals from individual voxels, thereby misrepresenting connectivity properties or network architecture^33–35^. Previous studies have also highlighted challenges in determining an optimal kernel size for spatial smoothing, particularly for resting-state fMRI.

Regarding the volume censoring and the secondary run selection, we simultaneously applied both FD and DVARS to scrub volumes containing large motions. Both FD and DVARS were defined by Power et al.^36^ as parameters of the head motion. FD is a parameter at the volume level, which is calculated using six motion parameters; three translations and three rotations. DVARS is a parameter at the voxel level and indicates the deviation of BOLD signals over the time series. As we wanted to censor volumes based on both FD and DVARS simultaneously, we averaged DVARS scores across all voxels for each volume in every run. For calculation of FD and DVARS, we developed a MATLAB script utilizing the “bramila_framewiseDisplacement” function (available at: https://github.com/spunt/bspm/blob/master/thirdparty/bramila/bramila_framewiseDisplacement.m) and “bramila_dvars” function (available at: https://github.com/spunt/bspm/blob/master/thirdparty/bramila/bramila_dvars.m) to censor volumes that exceeded the thresholds for either FD or DVARS and to establish regressor files for linear detrending. That is, only volumes that passed both thresholds of FD and DVARS could remain. For the calculation of DVARS, preprocessed functional files—with the first five volumes removed—were used instead of raw data to ensure more precise selection. The secondary run selection was based on the number of remaining volumes in each run, with the threshold of 103 volumes, equivalent to 4.5 min in the time course. All the runs that contained less than 103 volumes were excluded from further steps, resulting in 73 runs from 31 subjects (Table 1). Fourteen runs, including all the runs from two dogs (one male and one female), were discarded due to this selection process.

The latter two steps, the linear detrending and the band-pass filtering, were performed by CONN as denoising. We applied its default settings for both steps, except adding the created regressor files based on FD and DVARS as a confound for the linear detrending. By default, CONN performed linear regression as the linear detrending using an anatomical component-based noise correction procedure (aCompCor)^37^. This procedure included noise components from the cerebral white matter and cerebrospinal areas^38^, and a condition effect and added regressor files as confounds. In succession, CONN automatically performed the temporal band-pass filtering with the range of 0.008–0.09 Hz by default.

### ROI definition

**Fig. 1:**
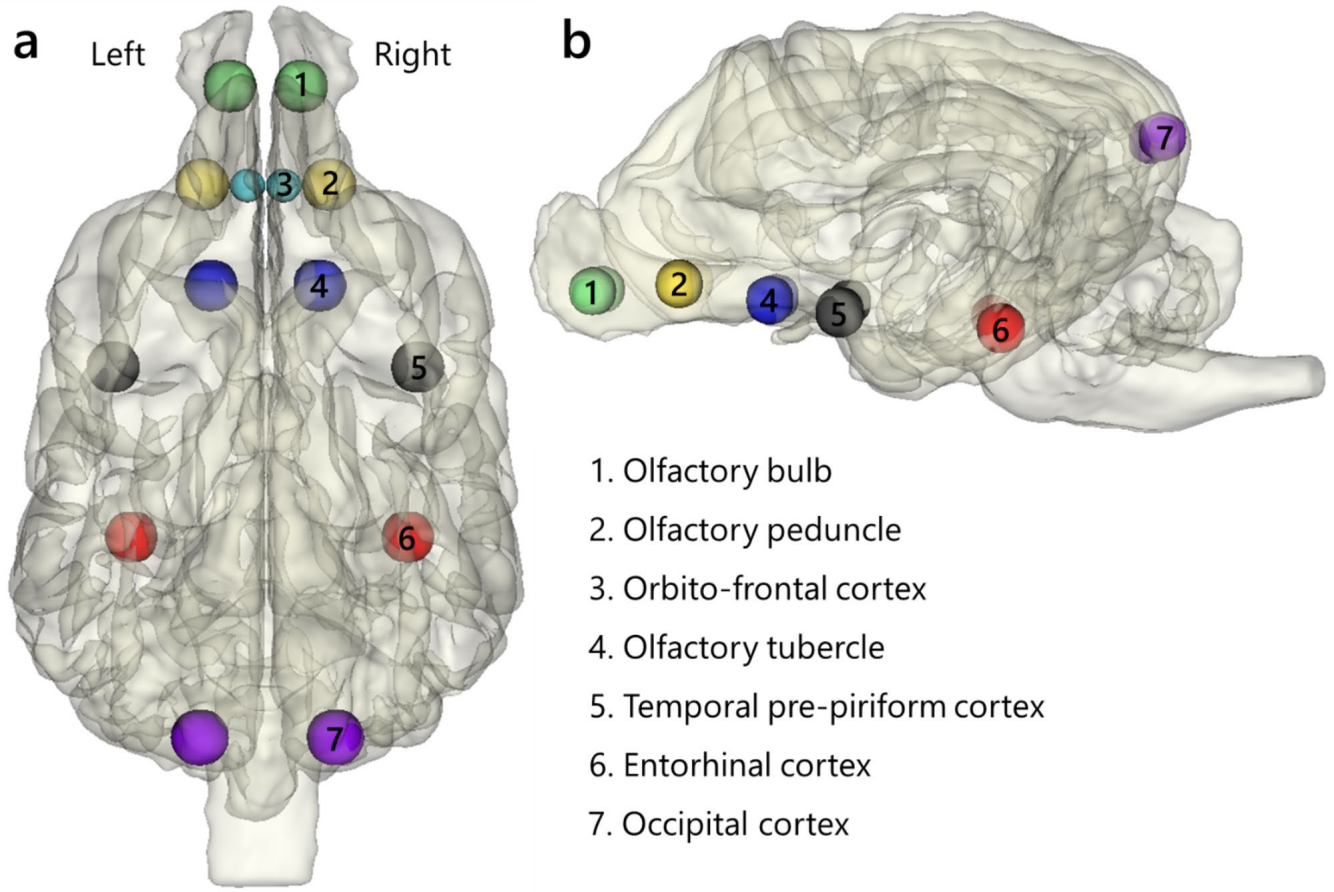
Dog olfactory network ROIs. Locations of ROIs in the brain. The size of ROIs was 3 mm in radius. The ROIs for the orbito-frontal cortex were 2 mm in radius. In the lateral view (**b**), the ROIs in the orbito-frontal cortices (3) are invisible because their locations are behind of ROIs in the olfactory peduncles (2) and their size is smaller than that of ROIs in the olfactory peduncles. The 3D brain figure with ROI was created using 3D Slicer (Version 5.2.1)^48^ (https://www.slicer.org) based on a publicly available template MRI image of a dog^32^ (https://figshare.com/s/628cbf7d4210271ffe70).

We defined ROIs for analysis to focus on specific areas in the brain. Spherical ROIs with a radius of 3 mm were created by the MarsBaR toolbox for MATLAB^39^. The reference image used for establishing these ROIs was the same preprocessed run (the first functional data in our dataset), and they were defined in seven regions in both hemispheres (Fig. 1): the olfactory bulb (X = $\:\pm\:$4, Y = 30, Z = −3), olfactory peduncle (X = $\:\pm\:$7, Y = 19, Z = −2), olfactory tubercle (X = $\:\pm\:$6, Y = 8, Z = −4), temporal pre-piriform cortex (X = $\:\pm\:$17, Y = −1, Z = −5), entorhinal cortex (X = 16/15, Y = −20, Z = −7), occipital cortex (X = $\:\pm\:$7, Y = −40, Z = −16), and orbito-frontal cortex (X = $\:\pm\:$2, Y = 19, Z = −3). Coordinates are reported in the in-house dog template space^32^. The total number of ROIs was 14. ROIs with a 2 mm radius were prepared specifically for the orbito-frontal cortices due to the narrowness of the region. Six regions, excluding the olfactory tubercle, were selected based on Andrews et al.^19^. This is the only study that investigated olfactory anatomical tracts from the olfactory bulb in dogs using diffusion tensor imaging (DTI). We excluded the medulla oblongata and stria terminalis from our ROIs due to their structures: the medulla oblongata contains neurons from multiple systems, including the olfactory system, while the stria terminalis is notably thin. These structural characteristics hindered the creation of accurate ROIs. In addition to the regions defined by Andrews et al.^19^, the olfactory tubercles were also added. These regions encompass the Islands of Calleja, a region reported to be involved in the affective valence processing of olfactory information in mice^40^. However, the Islands of Calleja region is smaller than our voxel size and are dispersed within the olfactory tubercle^41^. Therefore, we opted to generate ROIs for the olfactory tubercles encompassing the Islands of Calleja. To ensure that our ROIs captured signals predominantly from selected regions and not from outside the brain or adjacent areas, we positioned them approximately at the midpoint of these regions, guided by anatomical references^32,42–44^. We also confirmed that these ROIs seemed to be involved in olfactory processing using task-based fMRI studies^23,24^.

After creating ROIs, we assessed signal quality by calculating the temporal signal-to-noise ratio (tSNR) of all the ROIs in each preprocessed run. It was quantified as the mean signal over time divided by its standard deviation, calculated for each voxel and then averaged per ROI^45^.

**Table 1:** List of applied runs for analysis. The maximum number of available volumes is 132 without the first five volumes. Fourteen runs were excluded through the run selections, which included all runs from one female and one male dogs.

Subject ID	Run No.	No. of available volumes (excluding the first five volumes)	Subject ID	Run No.	No. of available volumes (excluding the first five volumes)
1	1	118	17	1	110
	2	121		2	117
2	1	124	18	1	112
	2	116	19	1	113
3	1	122		2	117
	2	126	20	1	124
4	1	130		2	127
	2	119		3	118
5	1	127	21	1	124
	2	126		2	121
6	1	116	23	1	120
	2	106		2	116
	3	109		3	125
7	1	121	24	1	124
	2	117		2	119
8	1	117		3	127
	2	123		4	124
9	1	118	25	1	115
	2	122		2	115
10	1	106		3	123
	2	115	26	1	124
	3	129		2	120
	4	127		3	126
11	1	120	27	1	123
	2	121		2	128
12	1	113		3	115
	2	118	28	1	122
13	1	120		2	116
	2	120	29	1	120
14	1	109		2	124
	2	126		3	119
15	1	116	30	1	125
	2	125		2	117
	3	107	31	1	121
16	1	127		2	124
	2	118	32	1	120
				2	113

### Analysis

Analysis was performed by CONN and Analysis ToolPak in Microsoft Excel. Since all the ROIs exhibited sufficient quality, we conducted ROI-to-ROI functional connectivity analysis with 14 ROIs. Functional connectivity was calculated for all pairs of ROIs, and a matrix of calculated scores was created per subject in the first-level analysis, and among subjects in the second-level analysis, applying the weighted General Linear Model (Weighted GLM) as the analysis type. Functional connectivity in the ROI-to-ROI connectivity matrix is defined as “Fisher-transformed bivariate correlation coefficient between a pair of ROIs BOLD timeseries”^37^. In the second-level analysis, multivariate parametric General Linear Model analysis was applied^46^. We utilized “ROI-level inference”, in which each ROI was treated as a “cluster”, to focus on the connectivity among individual ROIs. We applied the “All Subjects” effect on functional connectivity. By default, CONN applied F-statistics for “All Subjects”. In the “ROI-level inference”, an uncorrected ROI-level p-value and an FDR-corrected ROI-level p-value were utilized as the thresholds for connections and ROIs, with 0.05 and 0.01, respectively^46^. While examining individual effects of sex, age, and brain shape, we utilized the average score of all significant functional connectivity found in the all-subject analysis.

In terms of individual effects, we applied month age to measure participants’ age more accurately, considering that one year is more weighted for dogs compared with humans. Brain shape was quantified by the neuro-cephalic index, calculated as (brain width * 100) / brain length^47^. The length and width of each brain were measured using coordinates on the axial view of individuals’ anatomical T1-weighted images with 3D Slicer software (https://www.slicer.org)^48^. The measurement process was as follows: (1) identify the axial slice with the largest observable brain length or width; (2) define the starting point at one edge of the brain (e.g., for length, the anterior-most point; for width, the lateral-most point); (3) locate the opposite end of the starting point along the same axes (for length: X and Z axes; for width: Y and Z axes). The anterior end of the length was defined as the rostral end of the olfactory bulb, while the posterior end was defined as the caudal end of the cerebellum. The width was measured at the maximum distance between the lateral ends of the temporal lobes. The axial plane was defined to pass through both the anterior commissure and the posterior commissure to ensure they were on the same slice.

## Results

**Fig. 2:**
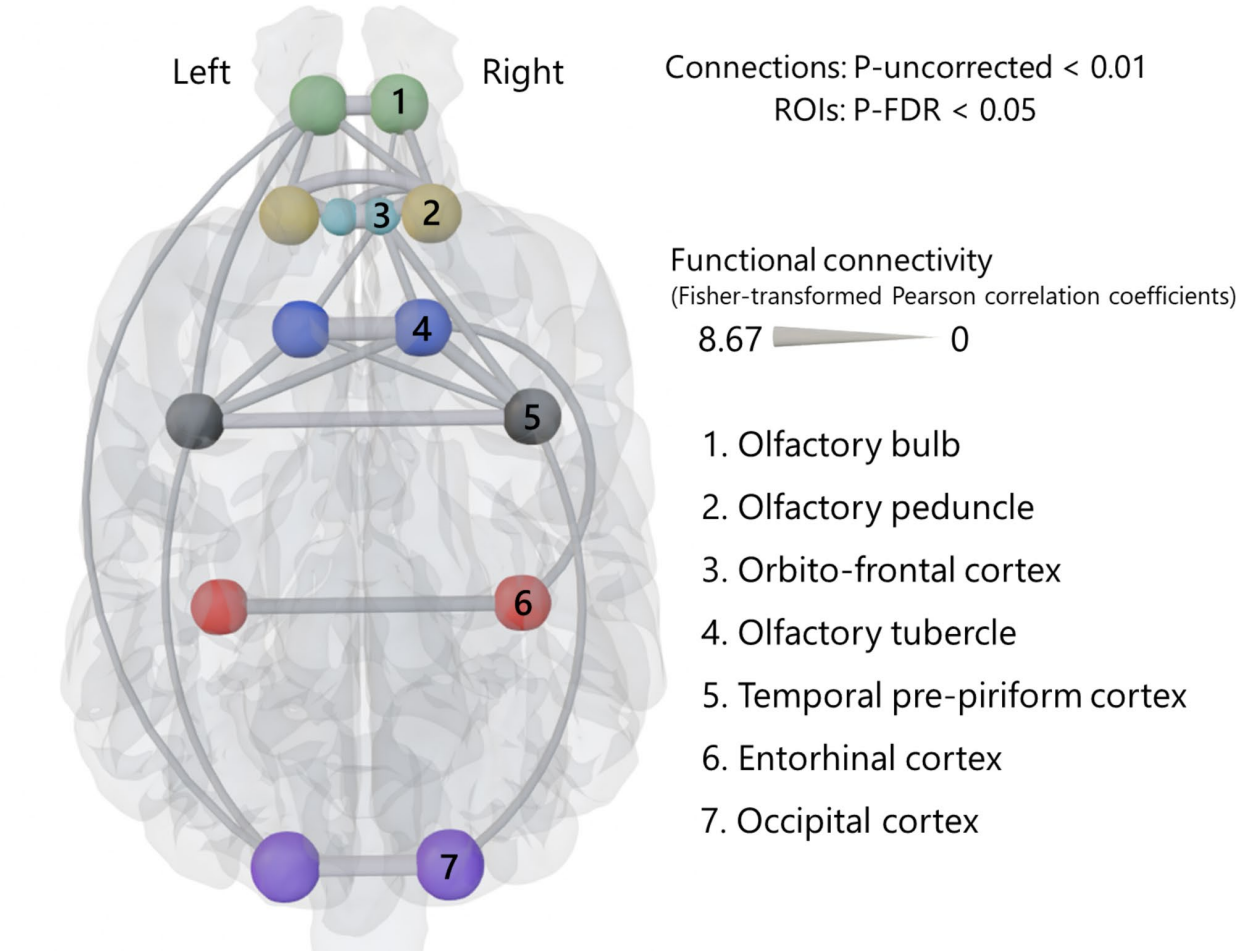
Resting-state functional connections across olfactory regions in the dog brain. Twenty-six significant connections across all 14 ROIs among all subjects, calculated by CONN. Calculated connections were Fisher-transformed Pearson correlation coefficients. The maximum value (8.67) is the highest correlation coefficient among found connections. The 3D brain image with ROIs and connections was created using Blender (Version 4.1, Blender Foundation, 2023) (https://www.blender.org) based on a publicly available template MRI image of a dog^32^ (https://figshare.com/s/628cbf7d4210271ffe70).

**Fig. 3:**
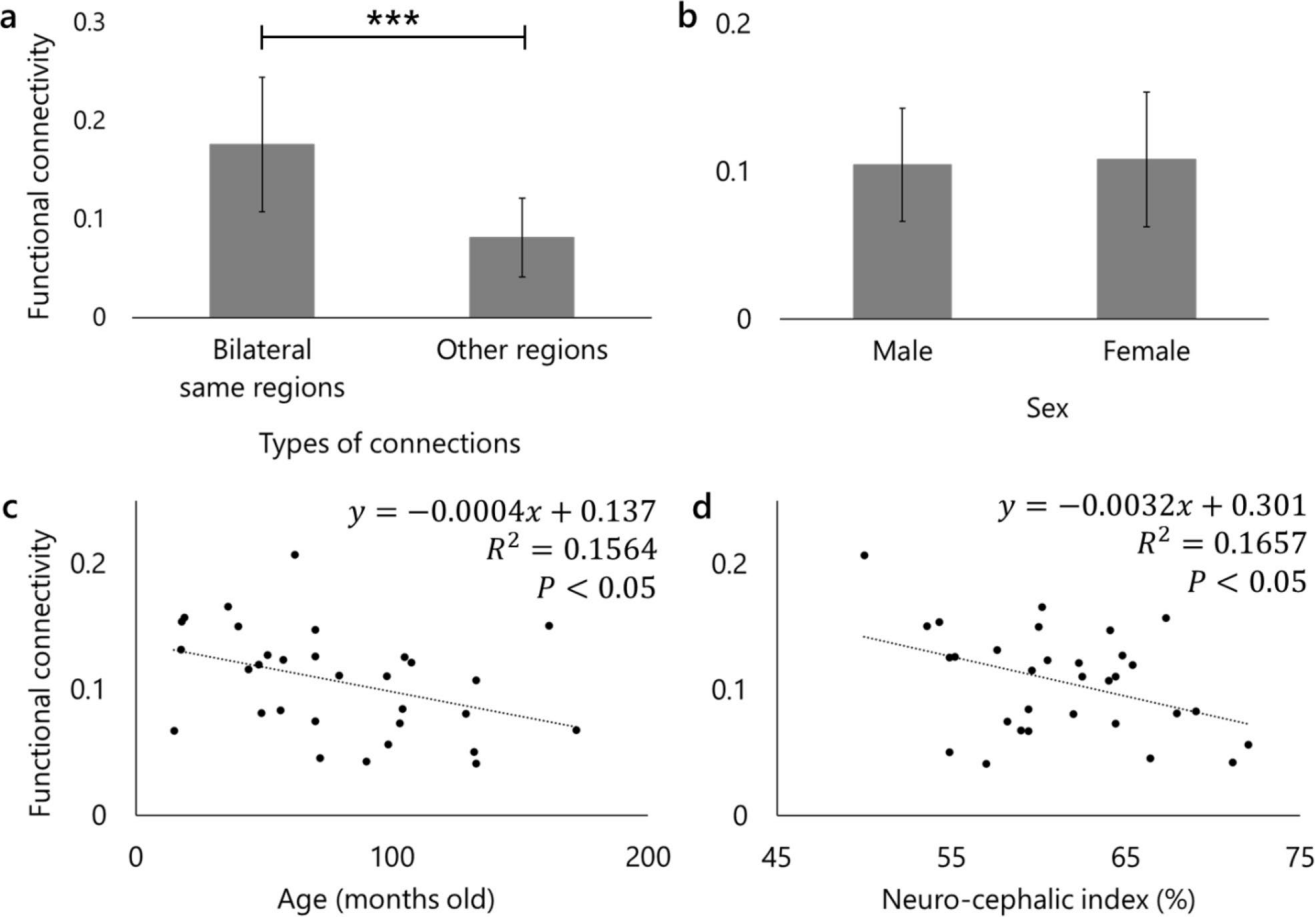
Results of bilateral connections and effects of individual characteristics on functional connectivity. The vertical axes of all the graphs represent the averaged functional connectivity of the 26 significant connections observed among all subjects (**a**, **c**, and **d**) or each sex (**b**). Error bars are standard errors of measurements. **a**: The result of two-samples t-test. The left bar indicates the averaged functional connectivity among all bilateral within-region connections in seven regions, while the right bar characterizes the average among connections between other regions. ***p < 0.001. **b**: The result of two-samples t-test on sex. The left bar shows the averaged functional connectivity among males, while the right bar is the average among females. The number of males and females were 15 and 16, respectively. **c**: The result of regression analysis on age. **d**: The result of regression analysis on brain shape. Neuro-cephalic index was quantified as (brain width * 100) / brain length^47^. A lower neuro-cephalic index indicates a rounder-shaped brain, while a high index illustrates a more elongated brain.

Functional connectivity network analysis with all subjects revealed 26 significant functional connections (seven bilateral within-region connections, eight connections in the right hemisphere, six connections in the left hemisphere, and five bilateral between-region connections) (Fig. 2). To provide an overall characterization of connection strengths in the olfactory network, we compared bilateral within-region connections, defined as connections between homogeneous regions from both hemispheres, with all other connections, using a two-sample t-test. Bilateral within-region connections were significantly stronger than connections between other regions (*p* < 0.001) (Fig. 3a). As for the effects of individual characteristics, averaged functional connectivity across the 26 significant connections was found not to be affected significantly by sex (*p* = 0.805) (Fig. 3b). For age and brain shape, in contrast, significant negative regressions were observed: R^2^ = 0.1564, *p* = 0.028 in age (Fig. 3c); R^2^ = 0.1657, *p* = 0.023 in brain shape (Fig. 3d). To exclude the possibility that the brain shape effect on functional connectivities was driven by the overrepresentation of two breeds of which we had multiple individuals in the sample (Golden Retrievers and Border Collies), we repeated the regression analysis after averaging the functional connectivities and neuro-cephalic indices within each of these breeds, thus using a single data point per breed. This follow-up analysis led to the same pattern of results, confirming the negative relationship between functional connectivity and brain shape (R^2^ = 0.2394, *p* = 0.033). We additionally performed a t-test comparing the average functional connectivity values of connections involving the olfactory bulb with those of all other significant connections, resulting in no significant difference (*p* = 0.24). These results indicate that younger dogs have stronger average functional connectivities in the olfactory network than older dogs, and dogs with more elongated brains (lower neuro-cephalic index) have stronger functional connectivity than those with rounder-shaped brains (higher neuro-cephalic index). Although we also analyzed at the level of individual connections, incorporating age, sex, and neurocephalic index as covariates, none of the covariates showed significant effects across all individual connections.

## Discussion

In this study, we investigated whether functional connectivity reflects individual variability of dogs’ olfactory performance, as well as whether resting-state functional connectivity in olfactorily relevant regions exhibits correspondence with previously described anatomical olfactory connectivity in dogs. We performed ROI-to-ROI functional connectivity analysis across 14 olfactory regions (seven in each hemisphere), utilizing a resting-state fMRI dataset, while we applied age, sex, and neuro-cephalic index as individual characteristics to test their effects on the connectivity. Among these regions, functional connections of varying strengths were identified in dogs, reflecting a pattern of only partial accordance with previously reported anatomical connectivities. Older and shorter-headed dogs exhibited weaker functional connectivities, suggesting that age and brain shape effects on functional connectivity strength may reflect behavioral patterns of olfactory performance. Finally, higher connectivities were observed within-region between hemispheres than between regions, paralleling reports from other species and reflecting the evolutionary anciency of such bilateral within-region connections.

The resting-state functional connectivity pattern found in the current study partially corresponds to the anatomical connectivity pattern reported by Andrews et al.^19^, and it also reveals additional connections not revealed by previous anatomical work. Although several studies reported that functional connectivity reflects underlying anatomical connectivity^49,50^, some studies demonstrated that strong functional connectivity could exist without structural connections or under weak structural connections^51,52^. Our results are in line with the latter findings. A remarkable difference between the present study and Andrews et al.^19 ^appeared in connections of the orbito-frontal cortex: in functional connectivity, the orbito-frontal cortex had connections not only with the piriform cortex and olfactory tubercle, which are known to be involved in higher processes (e.g.,^53,54^), but also with the olfactory bulb and olfactory peduncle, which are involved in the initial processing of olfactory information (e.g.,^55,56^). On the other hand, in the anatomical connectivity estimated by DTI and dissection, the frontal lobe, including the orbito-frontal cortex, was suggested as one of the terminations of the neural tracts initiated from the olfactory bulb^19^. Since the potential role of the orbito-frontal cortex is to generate olfactory consciousness, at least based on human studies^57,58^, we suggest that the functional connections found with initial processing regions are in line with this role. Therefore, our findings indicate that functional connectivity exists regardless of anatomical connectivity in canine olfactory regions and support previous findings about the central role of the orbito-frontal cortex in humans.

The higher functional connectivity observed in young dogs compared to old dogs suggests a deterioration of functional connectivity with aging. Age influences olfaction in various species, such as rodents (e.g.,^59^), monkeys (e.g.,^60^), humans (e.g.,^61^), and dogs. For example, geriatric dogs exhibit diminished accuracy in olfaction tasks compared to adult control dogs^8^, and aged dogs require a longer time to habituate to new smells^62^. In humans, aging is associated with alterations in functional connectivity within the olfactory network, such as between the medial frontal cortex and the angular gyrus^25,27^. The present finding is consistent with these results. However, our results appeared not to be specific to olfaction, as no significant difference was observed between connections involving the olfactory bulb and other significant connections, which is in line with previously observed age effect on functional connectivity in multiple brain networks^63^.

The finding of higher functional connectivity in dogs with more elongated brains compared to those with rounder-shaped brains is in accordance with several previous behavioral studies, although evidence on the relationship between olfactory ability and head shape is mixed. On the one hand, for example, previous behavioral studies demonstrated that breeds for scent work and longer-headed breeds showed higher performance in odor detection and discrimination tasks than shorter-headed breeds^4,5^. Our result is consistent with these findings. On the other hand, another behavioral study indicated an opposite pattern: short-headed pugs outperformed longer-headed German shepherds in odor-discrimination tasks^64^. In addition, findings of a relatively larger cribriform plate, the pathway of all olfactory nerve bundles from the periphery to the brain, in certain short-headed breeds compared to other breeds also indicate that some short-headed dogs may have morphological advantages for olfaction^17^. Future work is needed to better understand the potential causal relationships between functional olfactory connectivity and breed effects on dogs’ olfactory performance.

Finding no difference between males and females in functional connectivity across olfactory regions may be seen to contradict reports of sex differences in olfactory performance. Indeed, differences between males and females in olfactory capacities have been evidenced in dogs^6,9 ^as well as in other species, including humans (e.g.,^26,65,66^) and rats^67^. However, our finding of no sex difference in olfactory functional connectivity can be reconciled with these previous reports on olfactory sex difference. Specifically, it is also possible that behavioral sex effects on olfaction may rely on regions that are not part of the strictly defined olfactory network but may still be related to olfaction, such as the amygdala. In fact, previous works suggest an important role of the amygdala in behavioral and neural sexual dimorphism (e.g.,^68,69^). Our negative findings thus indicate that sex differences in olfactory performance, even if present, may not be reflected in resting-state olfactory functional connectivity.

In the current research, significantly higher within-region functional connections were observed between hemispheres than connections between other regions (regardless of them being unilateral or bilateral) in all the seven bilateral regions, suggesting the potential significance of bilateral within-region connectivities in the dog brain. High bilateral within-region functional connectivities have been previously observed across species. For example, in mice, strong bilateral within-region functional connections were observed across the entire brain^70^. In a human study focusing on olfactory regions, strong bilateral within-region connections were observed not only in healthy individuals but also in isolated congenital anosmia patients who are incapable of detecting any scents^71^. Our result, together with these studies in mice and humans, suggests that strong bilateral within-region connectivity is an evolutionarily conserved, default setting of the mammalian brain rather than specific to olfactory regions or olfactory experience.

The observed functional connectivity patterns were not identical between hemispheres. Hemispheric asymmetry in functional connectivity is considered to reflect functional specialization between hemispheres. For instance, hemispheric differences might indicate hemispheric dominance that certain networks exhibit larger clusters in one hemisphere, and both bilateral and unilateral components of the same functional area coexist^72^. However, in this study, none of the observed functional connections showed significant differences between hemispheres.

Although overall olfactory functional connectivity was affected by age and brain shape, none of the individual functional connections showed significant covariations with age, sex, or brain shape. One possible explanation is that the observed effects on each functional connection were too subtle to be detected by the statistical analysis. Alternatively, individual differences in functional connections might have been substantial. Regarding the latter possibility, we observed considerable variability in the strength of functional connections across individuals. To address this question, further research with rigorously controlled subject groups is required.

One limitation of the present study is the relative homogeneity of the brain shapes of the dogs. The range of neurocephalic indices was 50–72 with the lowest and highest cephalic indices from a Golden Retriever and a Swiss Shepherd, respectively. Indeed, we did not have typical brachycephalic breeds such as French Bulldogs or typical dolichocephalic breeds such as Greyhounds. It remains to be seen whether the correlation between brain shape and functional connectivity is generalized to dolichocephalic and brachycephalic dogs. Further investigation with dogs representing a wider range of brain shapes is recommended for future research. Moreover, as we investigated resting-state functional connectivity, it is possible that the observed connection patterns did not reflect olfactory processes specifically. Although we applied ROIs based on regions as determined by a single DTI tractography study^19^, the olfaction-related functional role of these connections will have to be confirmed in task fMRI studies. Nevertheless, most olfactory regions which we applied in the current study have already been reported in previous task fMRI studies on dog olfaction^23,24,73^.

## Conclusion

Together, the evidence presented in this study suggests a partial correspondence between anatomical and functional olfactory networks in the dog brain and general consistency between the effects of individual characteristics on behavior and functional connectivity in dogs’ olfaction. Future work will have to assess the potential of dog resting-state fMRI measures for informing the selection of suitable dogs for olfactory tasks.

## Data Availability

The dataset generated and analyzed in the current study are available from the corresponding author on reasonable requests.
